# Conditioned medium from human cord blood mesenchymal stem cells attenuates age-related immune dysfunctions

**DOI:** 10.3389/fcell.2022.1042609

**Published:** 2023-01-04

**Authors:** Bo Sun, Xianhui Meng, Yumin Li, Yanlong Li, Rui Liu, Zhongdang Xiao

**Affiliations:** ^1^ State key laboratory of bioelectronics, school of biological science and medical engineering, Southeast University, Nanjing, China; ^2^ Shandong Electric Power Central Hospital, Jinan, China; ^3^ Department of Genetic Engineering, College of Natural Science, University of Suwon, Hwaseong-si, South Korea

**Keywords:** cord blood, mesenchymal stem cells, immune regulation, oxidative damage, anti-aging

## Abstract

Aging is accompanied with progressive deterioration of immune responses and tissue’s function. Using 12-month-old mice as model, we showed that conditioned medium of human cord blood mesenchymal stem cells (CBMSC-CM) significantly reduced the population percentage of CD3^−^CD335^+^ NK and CD4^+^CD25^+^ regulatory T-cells in peripheral blood. The CBMSC-CM administration also increased naïve T-cells number and restored the ratio of naïve to memory T-cells in CD4^+^ T-cells population. These results indicated that CBMSC-CM improved the immune response efficiency of aged mice. Moreover, we also found CBMSC-CM treatment significantly reduced the number of senescenT-cells in kidney tissues. Finally, we demonstrated that CBMSC-CM remarkably attenuated hydrogen peroxide triggered T-cell response and ameliorated oxidative stress induced cellular senescence. All of these data suggest a prominent anti-aging effect of secretome of CBMSCs.

## Introduction

Aging profoundly increases the risk to many diseases such as cancer, diabetes, cardiovascular diseases and neurological disorders, etc. This is mainly due to the progressive decline of organs and tissues functions induced by accumulation of cellular damage (López-Otín et al., 2013; Barbosa et al., 2019; Hunt et al., 2019; Chakravarti et al., 2021). Particularly, most of the age-related diseases are associated with chronic inflammation, which can be partially attributed to the dysfunctional immune system with aging. The functional decline of immune system with aging, which is referred to as ‘immunosenescence’ not only increases the risk to infectious disease but also contributes to many degenerative diseases, such as neurodegeneration, cancer, cardiovascular and autoimmune disease (Fulop et al., 2018; Aiello et al., 2019; Chung et al., 2019; Duggal et al., 2019).

Mesenchymal stem cells (MSCs) are characterized as stromal stem cells or precursors with multipotent differentiation properties (Pittenger et al., 1999). They have been identified in different post-natal organs and tissues including brain, spleen, liver, kidney, lung, bone marrow, muscle, etc. (Meirelles et al., 2006). Although the exact functions of MSCs *in vivo* are still not clear, present studies suggest that they participate in tissue regeneration and play important roles in supporting neighboring progenitor cells and maintaining tissue homeostasis (Wang et al., 2020; da Silva Meirelles et al., 2008). The biological functions of MSCs also decline with age, which inevitably impairs the tissue regeneration capacity (Yang, 2018; Yang et al., 2018). Therefore, a rejuvenation of the MSCs’ activity is speculated as a promising strategy to improve functions of old tissues. To support this hypothesis, transplantation of young MSCs were found to slow the loss of bone density and even prolonged the life span of old mice (Shen et al., 2011; Lau et al., 2019). A recent study also demonstrated that transplantation of umbilical cord derived MSCs reversed cognitive aging of old mic (Cao et al., 2017). These studies indicate the anti-aging potential of MSCs. Considering the outstanding immune modulation functions of MSCs, whether MSCs could play a beneficial role in restoring age-related immune dysfunctions is an intriguing question that remains unanswered.

Extensive evidences showed that the biological functions of MSCs largely rely on their paracrine secretion property. MSCs secrete various bioactive compounds, including cytokines, remodeling factors and extracellular vesicles. Secretion of these factors is considered to be vital important to the trophic support and immune modulation functions of MSCs (Fu et al., 2017; Yuan et al., 2020). Therefore, it can be suggested that the secretome of MSCs may be responsible for their anti-aging effect.

In this study, we explored the anti-aging effect of MSCs derived conditioned medium (CM). The human umbilical cord blood (CB) has provided a fetal source of MSCs, which preserve a superior secretory function (Wagner et al., 2007). By a middle-aged mice model, we evaluated the effect of CBMSC-CM on the immunoregulatory functions and tissue aging of mice. *In vitro*, we also showed that CBMSC-CM attenuated hydrogen peroxide triggered T-cell response and protected stromal cells from oxidative stress induced cellular senescence.

## Results

### Conditioned medium from CBMSCs improved immunoregulatory functions of middle-aged mice

Our present study focused on the anti-aging effect of cytokine compounds from CBMSCs. The conditioned medium from CBMSCs (CBMSC-CM) was processed by ultracentrifuge to exclude the effect of extracellular vesicles. The CBMSC-CM was also concentrated by ultrafiltration to enrich the effective constituent and to reduce the interference of volume.

To determine whether CBMSC-CM could attenuate age-related immunosenescence *in vivo*, we evaluated the effect of CBMSC-CM on immune cell subpopulations of peripheral blood in a middle-aged mice model.

**FIGURE 1:**
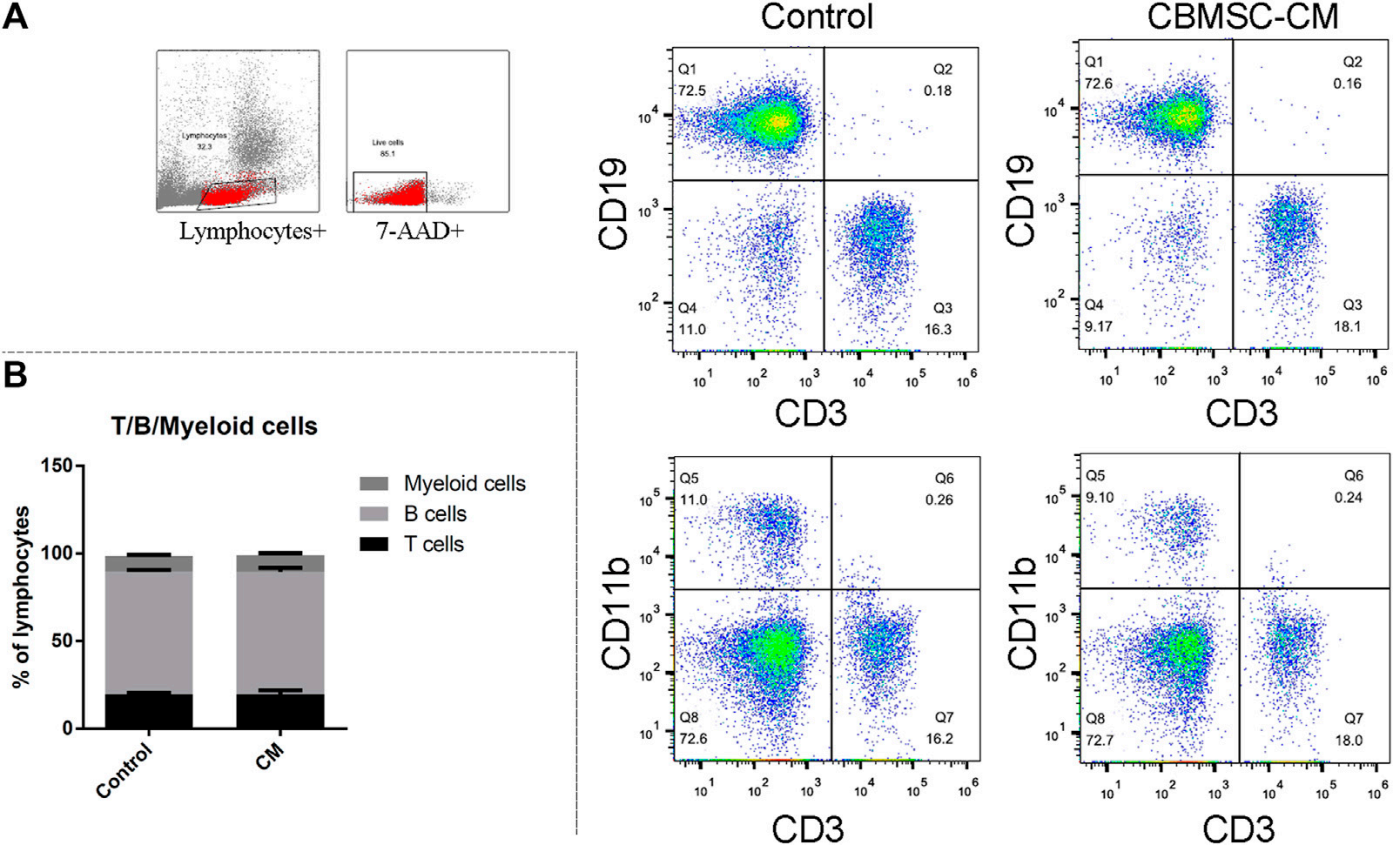
Flow cytometry analysis of T, B and myeloid cells in peripheral blood of old mice. **(A)** Representative detection of the T (CD3^+^CD19^−^), **(B)** (CD3^−^CD19^+^) and myeloid cells (CD3^−^CD11b^+^) of control and CBMSC-CM group. **(B)** Percentage of the T, B and myeloid cells was quantified. All values are expressed as mean ± SEM. (n = 5–8).

As the reduced levels of T (CD3^+^CD19^−^) and B (CD3^−^CD19^+^) cells population were taken as a hallmark of aging, we first detected the T, B and myeloid cells (CD3^−^CD11b^+^) percentage in the peripheral blood. There were no significant changes in the ratio of T, B and myeloid cells in control and CBMSC-CM treated group (Figures 1A,B). Moreover, the T and B-cell population from peripheral blood still maintained a high level in the old mice. This result may be explained by that the 12-month-old C57 Bl/6 mice were still in a middle-aged state, which did not come into a serious immunosenescence.

**FIGURE 2:**
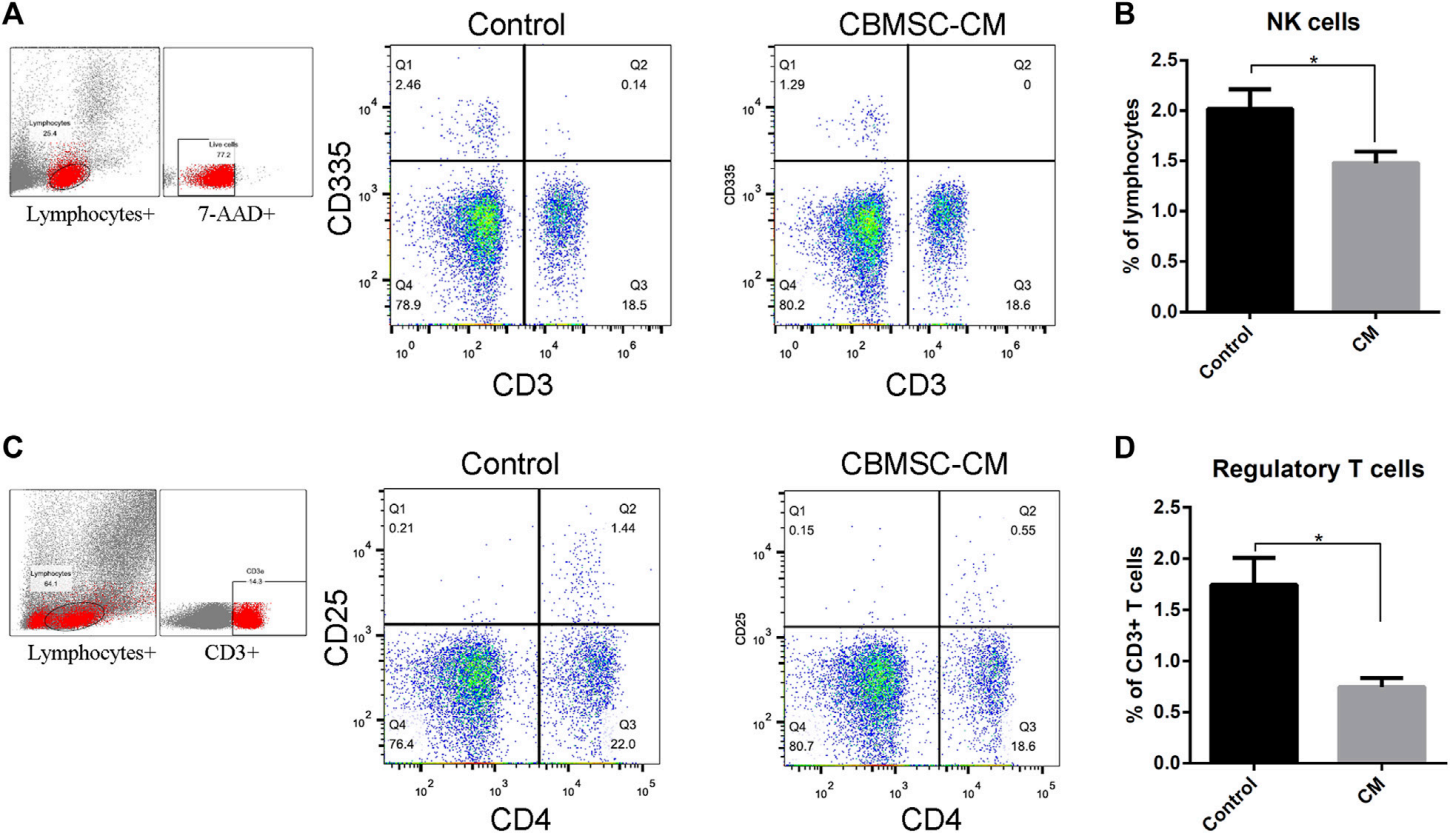
Flow cytometry analysis of NK cells and regulatory T-cells in peripheral blood of old mice. **(A)** Representative detection of the NK cells (CD3^−^CD335^+^) of control and CBMSC-CM group. **(B)** Percentage of the NK cells in lymphocytes was quantified (n = 5). **(C)** Representative detection of the regulatory T-cells (CD4^+^CD25^+^) of control and CBMSC-CM group. **(D)** Percentage of the regulatory T-cells in CD3^+^ T-cells was quantified (n = 7). All values are expressed as mean ± SEM. (n = 5–8), *p < .05.

Acting as an immune surveillance role against damaged cells such as senescenT-cells, natural killer cells (NK cells) have been reported changing in number and function with aging (Yang, 2018). We detected the level of CD3^−^CD25^+^ NK cells population in the peripheral blood of mice treated with control or CBMSC-CM. The result showed that CBMSC-CM treatment significantly decreased the percentage of NK cells population in peripheral blood (Figures 2A,B).

Regulatory T-cells (Tregs) maintains peripheral tolerance and act as critical negative regulators of inflammation (Vignali et al., 2008; Georgiev et al., 2019; Arroyo Hornero et al., 2020). Aging has been reported to be associated with increased Tregs numbers. Therefore, the level of Tregs was detected by measuring the CD4^+^CD25^+^ subpopulations in the CD3^+^ T-cells. The result showed that CBMSC-CM treatment significantly decreased the level of Tregs in peripheral blood (Figures 2C,D).

**FIGURE 3:**
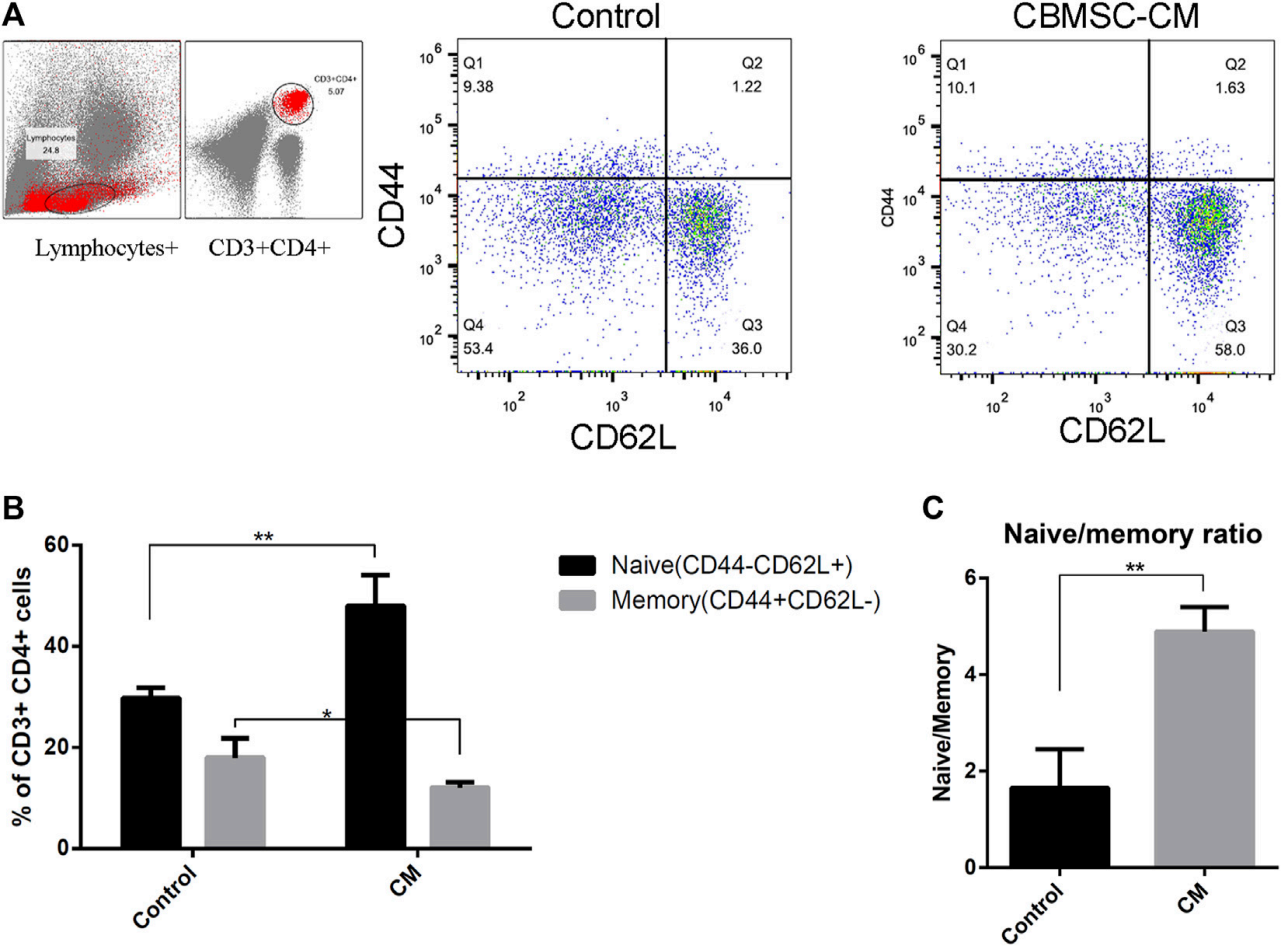
Flow cytometry analysis of naïve and memory T-cells in peripheral blood of old mice. **(A)** Representative detection of naïve (CD62L^+^CD44^−^) and memory (CD62L^−^CD44^+^) T-cells. **(B)** Statistical quantification of the naïve and memory T-cells in the CD3^+^CD4^+^ T-cells population (n = 3). **(C)** The ratio of naïve to memory T-cells were analyzed. All values are expressed as mean ± SEM. (n = 5–8), *p < .05, **p < .01.

The decrease of naïve/memory T-cell ratio with aging reflects the destruction of T-cell homeostasis. We found that CBMSC-CM treatment significantly increased the CD44^−^CD62L^+^ naïve T-cell population and decreased the CD44^+^CD62L^−^ memory T-cell population in the CD3^+^CD4^+^ subgroup of peripheral blood (Figures 3A,B). Therefore, compared to the control group, mice treated with CBMSC-CM maintained a higher ratio of naïve/memory T-cells in peripheral blood (Figure 3C).

### Conditioned medium from CBMSCs ameliorated kidney aging of middle-aged mice

**FIGURE 4:**
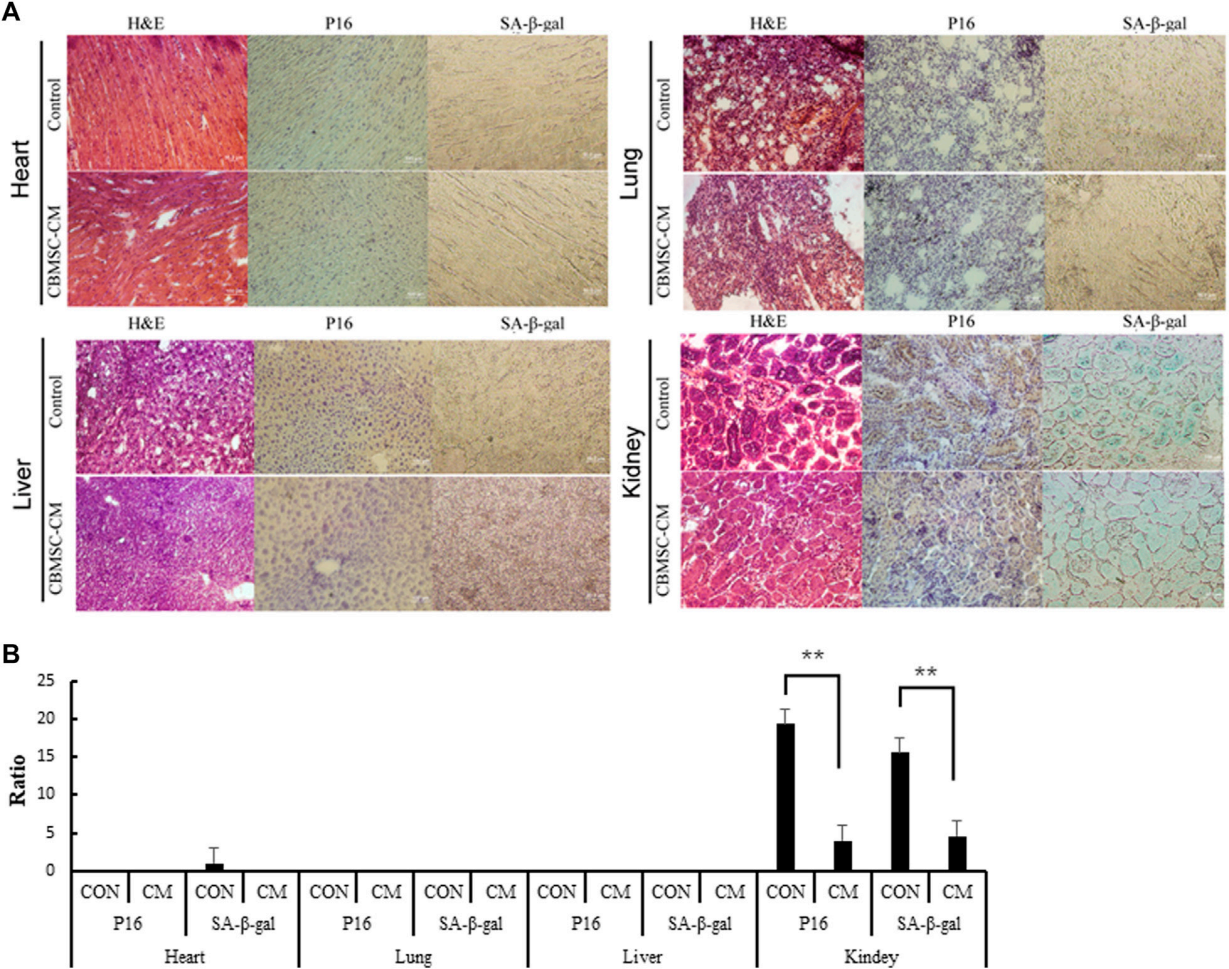
Detection of the senescenT-cells in tissues of old mice. **(A)** Heart, Liver, Lung and Kidney tissue samples were histologically analyzed by H&E staining. The senescenT-cells were detected by using P16 (Brown color) and SA-β-gal (Green color) as positive markers. **(B)** p16 positive cells and SA-β-GAL positive cells among the different groups were quantified.

To explore the effect of CBMSC-CM on other tissues in old mice, we further investigated the age-related phenotypes of representative tissues in old mice. Using P16 and SA-β-gal as markers, we labeled the senescenT-cells of heart, liver, lung and kidney tissues. The tissue cells in heart, liver and lung neither exhibited significant senescence phenotypes, nor showed any difference between control and experimental groups. In the kidney tissues we detected a high level of P16 expression and SA-β-gal staining, which suggested a profound aging of kidney. However, CBMSC-CM treatment significantly ameliorated the aging of kidney as detected by lower P16 expression and SA-β-gal staining (Figures 4A,B).

### Conditioned medium from CBMSCs attenuated hydrogen peroxide triggered T-cell response

**FIGURE 5:**
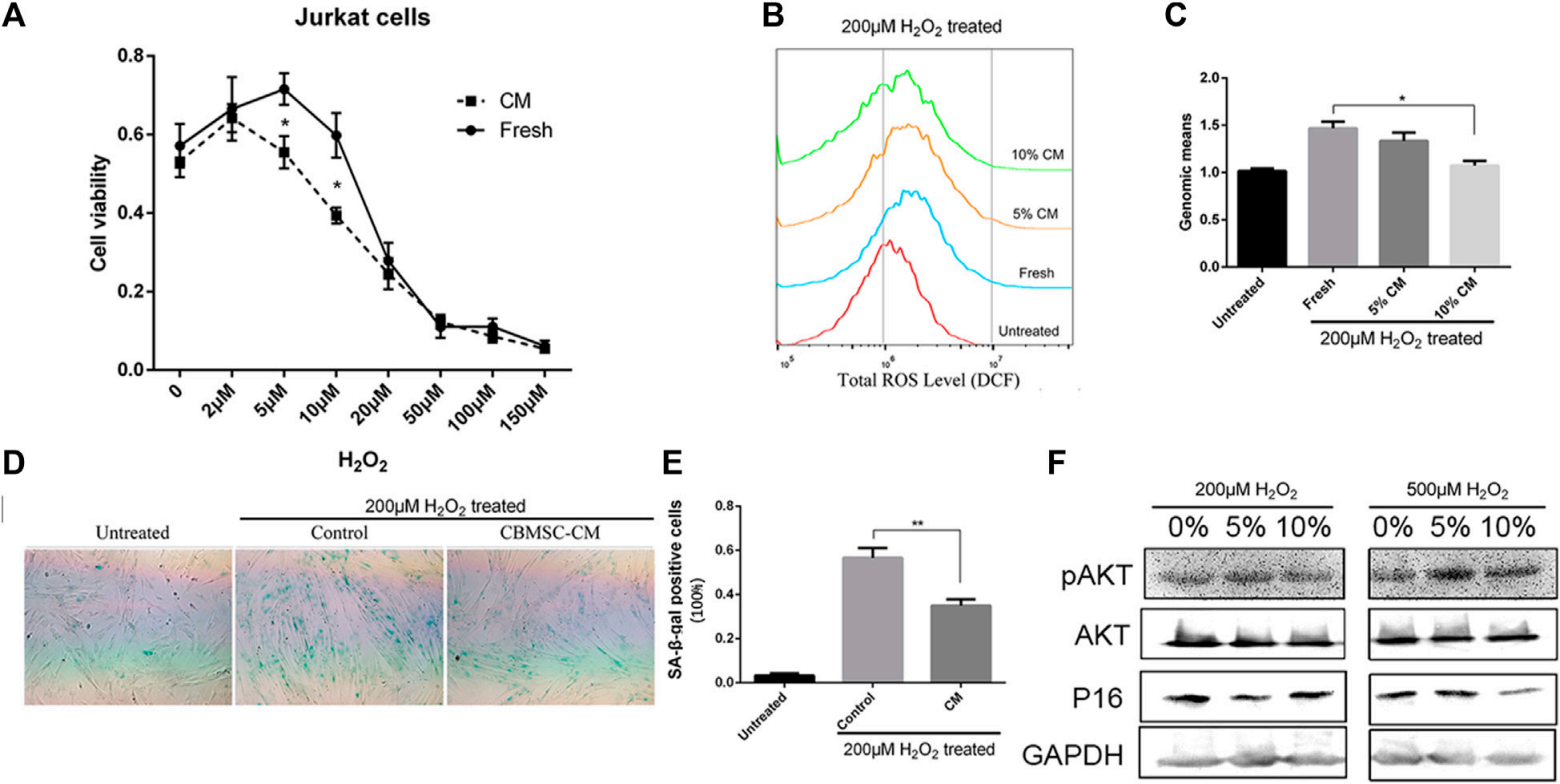
CBMSC-CM attenuated hydrogen peroxide induced oxidative stress. **(A)** Cell viability of JurkaT-cells was analyzed by CCK8 assay. The cells were pretreated with different concentration of H_2_O_2_ for 6 h and then cultured for another 18 h with fresh medium or medium supplemented with 10% CBMSC-CM. Data were from three independent experiments (n = 3). *, *p* < .05. **(B)** Representative result of the total ROS levels detected by flow cytometry. Cells were pretreated with 200 μM H_2_O_2_ and cultured with fresh medium or medium supplemented with 5% or 10% CBMSC-CM. **(C)** Total ROS levels were statistically analyzed according to the genomic means of fluorescence. Data were from three independent experiments (n = 3). *, *p* < .05. **(D)** Representative image of SA-β-gal staining (Green color) of the control and CBMSC-CM cultured cells pretreated with H_2_O_2_. **(E)** SA-β-gal positive cells were quantified from three independent experiments (n = 3). **, *p* < .01. **(F)** Levels of P16, pAKT and AKT in the CBMSCs pretreated with H_2_O_2_ were assessed by western blot (see also supplementary information1).

The increased production of hydrogen peroxide (H_2_O_2_) is a major clue of aging process and has been demonstrated to play an essential role in driving age-related inflammation and tissue cell damage (Hahn et al., 2017; Huang et al., 2018). We postulated that secretome released from young MSCs harbors factors protecting oxidative stress. Therefore, we next investigated whether CBMSC-CM treatment ameliorated H_2_O_2_ induced oxidative stress. The human T lymphocyte derived JurkaT-cells were exposed to different concentrations of H_2_O_2_ for 6 hours and cultured for another 18 h with medium containing 10% fresh control or CBMSC-CM. Interestingly, although high concentrations of H_2_O_2_ (>20 μM) significantly reduced the cell viability of JurkaT-cells, low concentrations of H_2_O_2_ (<10 μM) were shown to promote JurkaT-cells proliferation. However, when treated with CBMSC-CM, the promotion proliferation effect induced by H_2_O_2_ was remarkably repressed (Figure 5A). These results indicated that CBMSC-CM attenuated T-cell response triggered by H_2_O_2_.

### Conditioned medium from CBMSCs attenuated oxidative stress-induced cellular senescence

As in tissue stromal cells, a low concentration of H_2_O_2_ treatment is observed to trigger cellular senescence, we investigated whether CBMSC-CM played a role in protecting tissue stromal cells from oxidative stress induced cellular senescence. CBMSCs were pretreated with 200 μM H_2_O_2_ for 2 hours to expose to an oxidative challenge. Then the cells were incubated with fresh medium or medium supplemented with CM from young CBMSCs. After 3 days culture, the total reactive oxygen species (ROS) level of cells was measured by DCF probe. The results showed that pretreatment of H_2_O_2_ significantly increased the ROS level of CBMSCs. However, when treated with different concentrations of CBMSC-CM, there was a reduction trend of the ROS level in the cells and a 10% CBMSC-CM treatment showed significantly effective to reduce the ROS level of cells close to the untreated level (Figures 5B,C). We next evaluated the senescence state of cells by SA-β-gal staining. Pretreatment of H_2_O_2_ significantly increased the activity of SA-β-gal in the cells, which suggested a premature senescence. As we expected, treatment of 10% CBMSC-CM remarkably reduced the SA-β-gal activity in the cells (Figures 5D,E). As the PI3K/AKT signaling pathway provides important signal for the survival and growth of cells in response to oxidative stress, we evaluated the activity of serine/threonine kinase Akt. The result showed that CBMSC-CM treatment increased the level of pAkt while decreased the expression of P16 in response to H_2_O_2_ induction (Figure 5F). Therefore, CBMSC-CM treatment promoted the activity PI3K/AKT signaling pathway. These results implied that CBMSC-CM contained bioactive compounds that attenuated oxidative stress-induced cellular senescence.

## Discussion

In the present study, we found that the CBMSC-CM improved the immunoregulatory functions of old mice as evidenced by beneficial changes of NK cells, regulatory T-cells, and memory and naïve T-cells. We also confirmed that CBMSC-CM treatment reduced the rate of senescenT-cells in kidney tissue, which implied a delay of its aging process and improvement of its function. *In vitro*, we demonstrated that CBMSC-CM attenuated hydrogen peroxide induced T-cells response and prevented cells from oxidative stress induced cellular senescence. These results highlight the anti-aging activity exerted by CBMSC-CM.

Numerous studies have indicated that MSCs play essential roles in tissue regeneration processes. These data suggest that the activity of MSCs profoundly influences the neighboring stem cell niche and systemic milieu (Kuhn and Tuan, 2010; Asumda, 2013; Sui et al., 2019). It has become evident that the secretome of MSCs accounts for these biological functions. Although extracellular vesicles secreted by MSCs are taken as important factors, they have been depleted from the conditioned medium in this experiment. Whether treatment of extracellular vesicles plays roles in improving age-related functional deterioration is an intriguing question under investigation. Nevertheless, in this study we focused on the anti-aging functions of soluble proteins secreted by MSCs.

Age-related changes of the immune system involve in the innate part and the adaptive part. As representative of the innate part, NK cells contribute to the first line of defense against infections. Numerous studies confirmed that there is a significant increase in the percentage and/or absolute number of total NK cells with aging. This age-related increase in number can be taken as a compensation of the defective functions of NK cells (Huang et al., 2005; Le Garff‐Tavernier et al., 2010; Camous et al., 2012; Solana et al., 2014; Müller et al., 2019). In our study, an *in vivo* administration of CBMSC-CM significantly reduced the CD3^−^CD335^+^ NK cells. This indicated an improvement of innate immunity in the aged mice.

The age-related alteration of T-cell compartment could profoundly affect the adaptive immune response of aged population (Zhang et al., 2016). Although the immune modulation functions of MSCs often refer to their suppressive effect, our results found that the CD4^+^CD25^+^ Tregs in peripheral blood of aged mice were remarkably repressed by CBMSC-CM administration. Aging has been found associated with enhanced functions of Tregs, although the related mechanism remains unknown. Some studies support that the increased activity of Tregs with aging may be attributed to the long-term stimulation of inflammatory signals from damaged tissues. According to this theory, more Tregs are taken as a positive feedback to age-related degeneration (Madelung et al., 2015; Salminen, 2020). Despite its beneficial aspect, a high level of Tregs activity could attenuate the immune surveillance and increase body’s risk to cancer (Gliwiński et al., 2019). An intriguing question that remains to be uncovered is whether the increase number of Tregs came from the direct regulation by factors from CBMSC-CM, or is a response to improved inflammatory environment.

In our study, CBMSC-CM treatment significantly increased the level of naïve T-cells population in middle-aged mice. Age-related naïve T-cell defects has been taken as a prominent hallmark of immunosenescence and the reason can be attributed to thymic involution (Hahn et al., 2017). As the maturation site of T-cells, thymus undergoes profound atrophy with aging. This results in less efficient T-cell development and decreased emigration of naïve T-cells to the periphery. The decreased number of naïve T-cells disturbs the diversity of TCR repertoire, which impairing the response capacity of immune system to external infections. Studies have proposed that the functional decline of thymic stromal cells could account for thymic involution. It has been discovered that some crucial cytokines produced by thymic stromal cells such as G-CSF, GM-CSF, IL-7, M-CSF and SCF drive thymic involution (Lynch et al., 2009). Therefore, our results indicated that CBMSC-CM attenuated the thymic involution in aged mice and this may be due to the improved thymus microenvironment.

Our results showed that the tissue cells in heart, liver and lung exhibited no significant senescence phenotypes. This may be the reason that the 12-month-old mice were still in a middle-aged state and the low aging state of heart, liver and lung tissues counteracted the effect of CBMSC-CM. Nevertheless, we have found that CBMSC-CM administration significantly reduced the number of senescenT-cells in kidney. The immune cells are responsible for the clearance of senescenT-cells of old tissues. The accumulation of senescenT-cells in organs and tissues can be partially explained by the reduced efficiency of senescent cell clearance by immune system (Ovadya et al., 2018; Gasek et al., 2021). Therefore, the more efficient immune response improved by CBMSC-CM may be responsible for the ameliorated kidney aging.

ROS related oxidative damage has been taken as a major threat of cell and tissue activity. Our *in vitro* assay indicated that the anti-aging effect of CBMSC-CM could be largely attributed to the improved cellular resistance to oxidative stress. In the immune system, H_2_O_2_ can act as a secondary messenger in the initiation and amplification of signaling at the antigen receptor (Di Marzo et al., 2018). Exposure to low concentration of H_2_O_2_ mimicked the effect of antigen and triggered proliferation of JurkaT-cells. However, treatment of CBMSC-CM reduced the response sensitivity of T-cells to H_2_O_2_. Given this result, CBMSC-CM may prohibit T-cells exhaustion from persistent antigen stimulation and improve chronic infections. Furthermore, we also demonstrated that CBMSC-CM significantly attenuated oxidative stress induced cellular senescence and the related mechanism involved in the activation of the AKT signal.

In summary, our study provided the evidences that soluble factors secreted by CBMSCs ameliorate oxidative damage and attenuate the aging process of old tissues. These results emphasized the potential application of conditioned medium from MSCs in the therapy of aging and age-related diseases. The use of the conditioned medium as a cell-free strategy for therapy would significantly reduce the risk of allogeneic transplantation of MSCs.

## Materials and methods

### Cell culture

Human umbilical cord blood samples were kindly donated from Jiangsu Yinfeng Biological Technology CO. Ltd. (Nanjing, Jiangsu, China). Methods used in this study were carried out according to the relevant regulations and guidelines. Human mesenchymal stem cells from umbilical cord blood (CBMSC) were prepared as previously described (Meng et al., 2016). Briefly, the mononuclear cells were first separated by density-gradient centrifugation from umbilical cord blood. Then they were seeded into six-well plates at a density of 5×10^6^/well and cultured in DMEM/F12 medium (Wisent, Nanjing, JS, China) supplemented with 10% FBS (Wisent), 1% Penicillin/Streptomycin (Hyclone, Logan, UT, United States) and 2 mM L-glutamine at 37°C in 5% CO_2_. JurkaT-cells were maintained in RPMI1640 medium supplemented with 5% FBS, 1% Penicillin/Streptomycin (Hyclone) and 2 mM L-glutamine at 37°C in 5% CO_2_ at a density of 10^6^ cells per ml.

### Conditioned medium collection

To collect conditioned medium from CBMSCs (CBMSC-CM), CBMSCs before passage eight were cultured until 70% confluence. Then the cells were washed twice with PBS and further cultured with fresh DMEM/F12 medium for another 48 h. Thereafter, the supernatant was collected. To remove cellular debris and extracellular vesicles, the supernatant was first centrifuged at 12,000×*g* for 30 min, and then centrifuged at 100,000×*g* for 70 min. Finally, the obtained supernatant was concentrated 15-fold using a 3 KDa ultrafiltration membrane (Millipore, Bedfored, MA, United States) and stored at −80°C until use.

### Mice model

C57BL/6 male mice at 12-month-old were used in this experiment. The mice were divided into control (n = 7) and CBMSC-CM treatment (n = 7) groups. They were intravenously injected with 200 μl concentrated CBMSC-CM or control medium every 3 days for 1 month. Then, the mice were fed for another month until the following test.

### Flow cytometry analysis

For the flow cytometry assay, the cells were assayed for fluorescence using an Accuri C6 cytometer (BD Bioscience, Ann Arbor, MI, United States). Data were analyzed using the Flowjo software (Tree Star, Inc. San Carlos, CA, United States).

To evaluate the immune cell phenotypes of old mice, the peripheral blood of mice was collected by submandibular bleeding. To analyze the expression of T-cells, B-cell, myeloid cells, natural killer cells (NK cells), regulatory T-cells (Tregs), CD4^+^ naïve T-cells and memory T-cells, the blood samples were first blocked by purified anti-mouse CD16/32 antibody (Biolegend, San Diego, CA, United States) for 10 min on ice. Then they were stained with fluorescence-conjugated anti-mouse antibodies against CD3 (-PE), CD4 (-FITC), CD11b (-APC), CD19 (-FITC), CD335 (FITC), CD25 (-APC), CD44 (-PerCP-Cy5.5) and CD62L (-APC, all from Biolegend) for 20 min at room temperature in the dark and washed with PBS for two times. Thereafter, the blood samples were lysed by red blood cell lysis buffer (eBioscience, San Diego, CA, United States) for 8 min at room temperature in the dark, washed with PBS for two times. Finally, the samples were detected by flow cytometry.

### Histological analysis

Tissues were embedded in OCT compounds and snap-frozen. Then the tissues were processed into 8-μm frozen sections using a cryotome and subsequently fixed with 100% acetone for 2 minutes. The sections were stored in −80°C for further analysis. Histology examination was performed by staining the sections with hematoxylin and eosin (H&E) (Jiancheng Bio, Nanjing, JS, China).

### Immunohistochemistry

Tissue sections were treated with .5% Triton-X100 in TBS buffer for 30 min and then washed with TBS buffer. The sections were blocked with 10% FBS and 1% BSA for 2 hours at room temperature. Then they were incubated with P16 antibody (1:50, Santa Cruz Biotech, Santa Cruz, CA, United States) or SA-β-gal (1:50, Santa Cruz Biotech, Santa Cruz, CA, United States) at 4°C overnight. After treated with .3% H_2_O_2_ in TBS buffer for 15 min, the sections were incubated with peroxidase-conjugated secondary antibody (Kangcheng Bio, Shanghai, China) for 1 hour at room temperature. Finally, the sections were visualized using a DAB kit (Beyotime Bio, Shanghai, China) and assessed by microscopy.

To quantifying the number of p16 positive cells and SA-β-GAL positive cells in the tissue slide, five visual field were randomly selected on each slide and the ratio of positive cells in each visual field were recorded. The ratio in each visual field was obtained as the following formula: Ratio of positive cells = Total number of positive cell/Total number of cells. The total positive ratio of one slide were calculated as: Ratio of positive cell on each slide = The sum of the ratio of the 5 field/5.

### Hydrogen peroxide treatment

JurkaT-cells were pretreated with hydrogen peroxide (H_2_O_2_) with gradient concentrations (0 μM, 5 μM, 10 μM, 15 μM, 20 μM, 50 μM, 100 μM, 150 μM) for 6 hours. After discarding the old medium, the cells were resuspended with fresh medium and seeded into 96 well plate at a density of 2×10^4^/well. Then the cells were added with 10 μl control or CBMSC-CM medium (10%) and incubated for another 18 h.

CBMSCs were seeded into six-well plates until 50% confluence. Then they were treated with 200 μM or 500 μM hydrogen peroxide (H_2_O_2_) for 2 hours. After discarding old medium, the cells were washed three times with PBS. Finally, they were cultured with fresh medium containing CBMSC-CM or control medium and cultured for another 3 days.

### Cell viability assay

The cell viability of JurkaT-cells were assayed by CCK8 reagent (Beyotime, Shanghai, China). Cells cultured in 96 well plate were treated with the indicated reagents and incubated for 1 h. Cell viability was determined by the absorbance at 450 nm wavelength (with reference wavelength of 650 nm) measured using a microplate absorbance reader (Biotek, Winooski, VT, United States).

### Reactive oxygen species detection

CBMSCs cultured in six-well plates were incubated with 5 μM DCFH-DA (Beyotime, Shanghai, China) at 37°C for 20 min. Then the cells were washed three times with fresh medium, digested with trypsin, and collected. Finally, the fluorescence was detected by flow cytometry.

### SA-β-gal staining

SA-β-gal staining was performed using a SA-β-gal staining kit (Beyotime). Briefly, the cells or tissue sections were fixed using the fixation buffer for 15 min and then incubated with the staining solution at 37°C overnight. The cells or sections were observed by microscopy. The ratio of positive cells was determined by counting the blue cells *versus* total cells.

### Western blot

Total cell proteins were extracted using a total protein extraction kit (Sangon Biotech, Shanghai, China), resolved by 12% SDS-PAGE and transferred to PVDF membranes (Roche Diagnostics, Mannheim, Germany). Detection of protein expression was performed using anti-phospho-AKT1 (Ser473) (Thermo Fisher Scientific, Waltham, MA, United States), anti-AKT Pan (Thermo Fisher Scientific) and anti-GAPDH (Santa Cruz Biotechnology, Santa Cruz, CA, United States).

### Statistical analysis

All data were expressed as the means ± SEM. Statistical analysis was performed using Graphpad prism (Graphpad Software. San Diego, CA, United States) and a two-tail Student’s t-test was used to compare two groups. A *p*-value of less than .05 was considered statistically significant.

## Data Availability

The original contributions presented in the study are included in the article/Supplementary Material, further inquiries can be directed to the corresponding authors.
