# Supplementary material for: Conditioned medium from human cord blood mesenchymal stem cells attenuates age-related immune dysfunctions
Source: Front Cell Dev Biol. 2023 Jan 4;10:1042609. doi: 10.3389/fcell.2022.1042609 (PMC9846238; doi:10.3389/fcell.2022.1042609)
Supplement: Supplementary file 1 [file DataSheet1.PDF]

supplementary information

The protein blot images in Figure 5F were collected from different gels or fields.

Supplementary Figure 1 provide the full length gel where the corresponding pAKT brands(red boxes and arrows) were cropped in Figure 5F.

Supplementary Figure 2 provide the full length gel where the corresponding p16 brands(red boxes and arrows) were cropped in Figure 5F.

Supplementary Figure 3 provide the full length gel where the corresponding total AKT brands(red boxes and arrows) and corresponding GAPDH(yellow boxes and arrows) were cropped in Figure 5F.

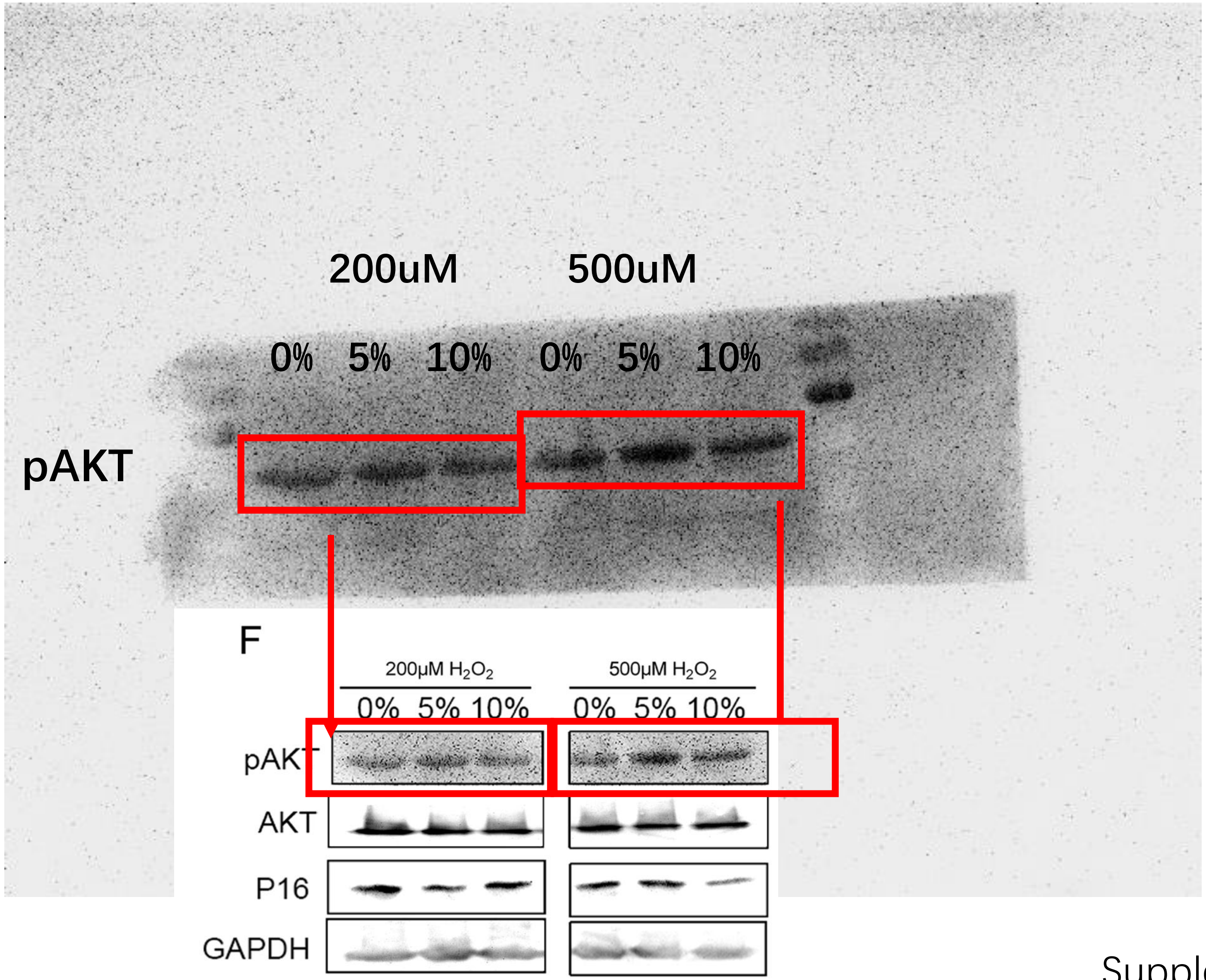

Supplementary Figure1

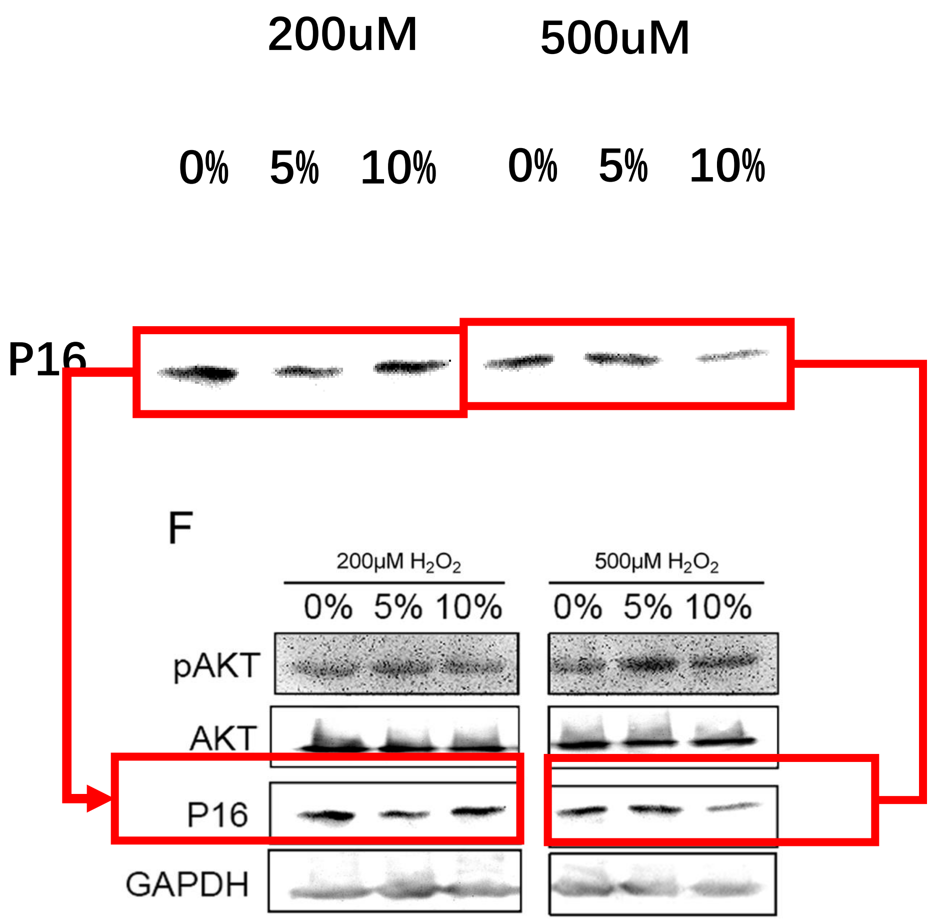

Supplementary Figure2

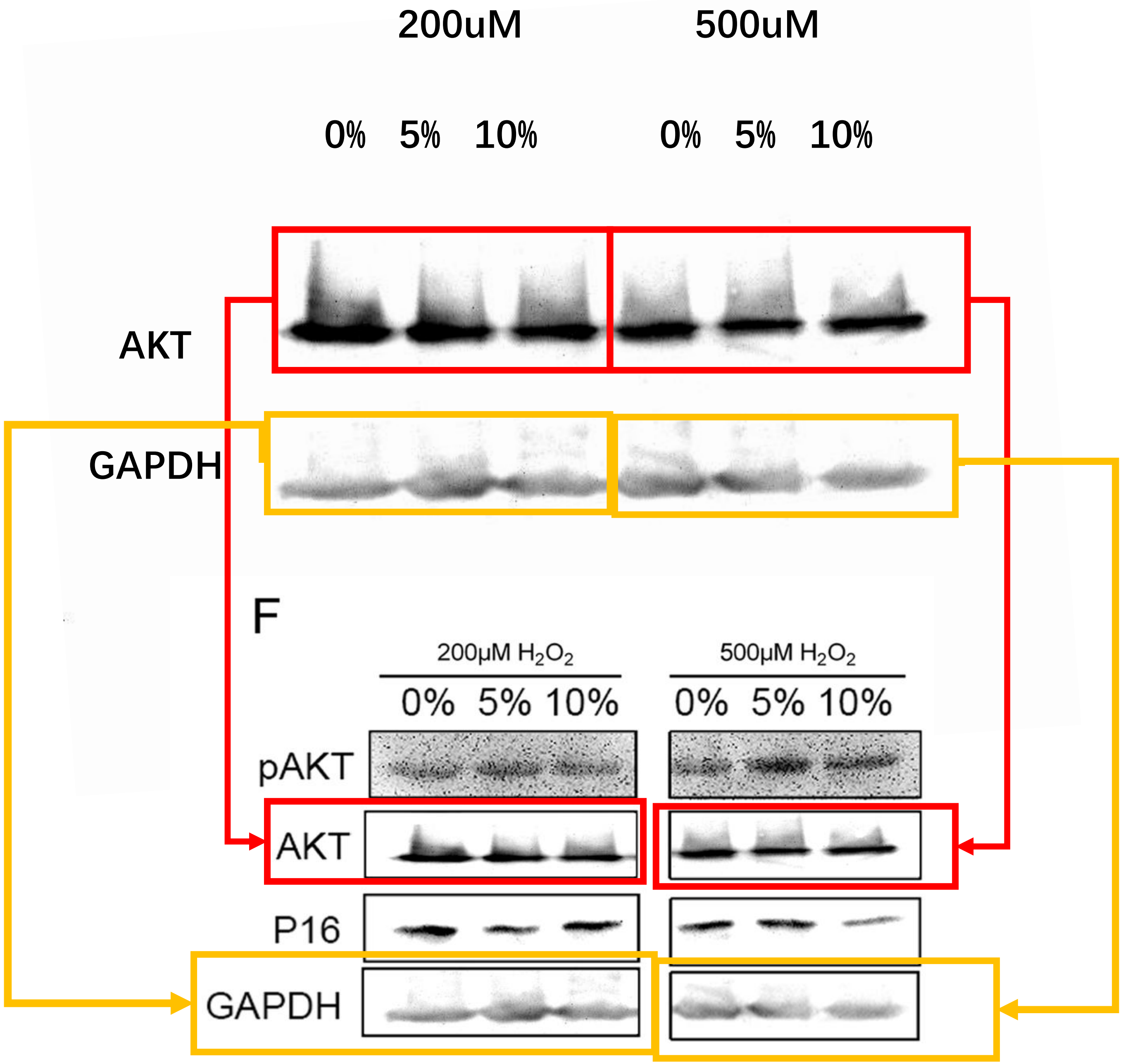

Supplementary Figure3
